# Perspectives of primary health care staff on the implementation of a sexual health quality improvement program: a qualitative study in remote aboriginal communities in Australia

**DOI:** 10.1186/s12913-018-3024-y

**Published:** 2018-04-02

**Authors:** Belinda Hengel, Stephen Bell, Linda Garton, James Ward, Alice Rumbold, Debbie Taylor-Thomson, Bronwyn Silver, Skye McGregor, Amalie Dyda, Janet Knox, Rebecca Guy, Lisa Maher, John Martin Kaldor, on behalf of the STRIVE Investigators, John Kaldor, James Ward, Alice Rumbold, Rebecca Guy, Robyn McDermott, Lisa Maher, Steven Skov, John Boffa, Donna Ah. Chee, Skye McGregor, Bronwyn Silver, Debbie Taylor-Thomson, Linda Garton, Belinda Hengel, Janet Knox, Amalie Dyda, Mathew Law, Christopher Fairley, Basil Donovan, David Glance

**Affiliations:** 1 Apunipima Cape York Health Council, PO Box 12045, Earlville, Cairns, Qld 4870 Australia; 2 Kirby Institute, UNSW Sydney, Wallace Wurth Building, Kensington, NSW 2052 Australia; 3 NT Department of Health, Sexual Health and Blood Borne Virus Unit, Casuarina, NT 0811 Australia; 4 South Australian Health and Medical Research Institute, North Terrace, Adelaide, SA 5000 Australia; 5 Menzies School of Health Research, Darwin, NT 0810 Australia; 6 Robinson Research Institute, University of Adelaide, Adelaide, SA 5006 Australia; 7 Lismore Sexual Health Service, NSW Health, Sydney, NSW 2480 Australia; 8 Flinders University, Adelaide, SA 5000 Australia; 9 Centre for Social Research in Health, UNSW Sydney, Sydney, NSW 2052 Australia; 10 Melbourne School of Population and Global Health, University of Melbourne, Victoria, 3010 Australia; 11 Central Australian Aboriginal Congress, Alice Springs, NT 0870 Australia

**Keywords:** Sexual health, Continuous quality improvement, Normalisation process theory, Aboriginal

## Abstract

**Background:**

Young people living in remote Australian Aboriginal communities experience high rates of sexually transmissible infections (STIs). STRIVE (STIs in Remote communities, ImproVed and Enhanced primary care) was a cluster randomised control trial of a sexual health continuous quality improvement (CQI) program. As part of the trial, qualitative research was conducted to explore staff perceptions of the CQI components, their normalisation and integration into routine practice, and the factors which influenced these processes.

**Methods:**

In-depth semi-structured interviews were conducted with 41 clinical staff at 22 remote community clinics during 2011–2013. Normalisation process theory was used to frame the analysis of interview data and to provide insights into enablers and barriers to the integration and normalisation of the CQI program and its six specific components.

**Results:**

Of the CQI components, participants reported that the clinical data reports had the highest degree of integration and normalisation. Action plan setting, the Systems Assessment Tool, and the STRIVE coordinator role, were perceived as adding value to the program, but were less readily integrated or normalised. The remaining two components (dedicated funding for health promotion and service incentive payments) were seen as least relevant. Our analysis also highlighted factors which enabled greater integration of the CQI components. These included familiarity with CQI tools, increased accountability of health centre staff and the translation of the CQI program into guideline-driven care. The analysis also identified barriers, including high staff turnover, limited time involved in the program and competing clinical demands and programs.

**Conclusions:**

Across all of the CQI components, the clinical data reports had the highest degree of integration and normalisation. The action plans, systems assessment tool and the STRIVE coordinator role all complemented the data reports and allowed these components to be translated directly into clinical activity. To ensure their uptake, CQI programs must acknowledge local clinical guidelines, be compatible with translation into clinical activity and have managerial support. Sexual health CQI needs to align with other CQI activities, engage staff and promote accountability through the provision of clinic specific data and regular face-to-face meetings.

**Trial registration:**

Australian and New Zealand Clinical Trials Registry ACTRN12610000358044. Registered 6/05/2010. Prospectively Registered.

**Electronic supplementary material:**

The online version of this article (10.1186/s12913-018-3024-y) contains supplementary material, which is available to authorized users.

## Background

Young people living in remote Aboriginal and Torres Strait Islander (hereafter referred to as ‘Aboriginal’) communities in central and northern Australia experience disproportionately high rates of bacterial sexually transmitted infections (STIs), particularly *Chlamydia Trachomatis*, *Neisseria Gonorrhoea* and *Trichomonas Vaginalis* [1]. A recent study across 67 remote communities reported that nearly 50% of young women aged 16–19 years at these remote clinics had at least one of these three infections [2], despite decades of programmatic and policy initiatives aimed at improving prevention and treatment [3].

A systematic review of STI programs within Australian remote primary care settings suggested that the comprehensive application of clinical best practice, including increased screening for STIs, timely treatment, 3 month test for reinfection and partner notification in primary care settings may help to reduce rates of STIs [4]. Continuous quality improvement (CQI) is an approach that has been widely used to enhance the implementation of best practice in health care delivery [5]. CQI is the cyclical, systematic, objective measurement of health care delivery aimed to develop strategies to work toward improving outcomes for the population [6]. CQI has been used internationally [5], and in Australian remote settings primarily for chronic disease management [7]. Within the context of chronic disease CQI in Australia, successful outcomes have been reported as, among other factors, reliant on the clinic staff exhibiting shared CQI goals, operationalising CQI tools and embedding CQI activities into routine clinical practice [8]. The STRIVE (STIs in Remote communities, ImproVed and Enhanced primary care) trial was designed to test whether a CQI approach could improve the uptake of sexual health clinical best practice in remote communities. The trial, described in detail elsewhere [9], aimed to assess if a sexual health CQI program (Table 1), consisting of clinical data reports, a Systems Assessment Tool, action planning, an external, visiting coordinator, health promotion funding and incentive payments, could assist clinics to reach best practice targets for the testing and management of STIs and, in turn, reduce population prevalence of infection. To our knowledge there has been no systematic evaluation of the degree of normalisation of a sexual health CQI program, within Australia or in other international settings. Therefore, as part of the trial, we conducted qualitative research with clinical staff to gain insight into staff perceptions of the sexual health CQI components, their normalisation and integration into routine practice, and the factors which influenced these processes (Additional file 1).

**Table 1:** STRIVE CQI program

STRIVE CQI component	Description
Clinical data report	• De-identified data extracted from (i) participating laboratories and (ii) electronic medical records • Both data sets used to ensure data quality • 6 monthly clinic-specific clinical data reports presented to staff at a face-to-face visit • Visual format provided clinic staff with insights into their local clinical practice associated with the STRIVE ‘best practice targets’
Systems Assessments Tool	• Using the Systems Assessment Tool developed by other Australian CQI programs [7], STRIVE developed a tool specific to sexual health • Encompassed six components which impacted on the systematic delivery of sexual health care • Staff self-rated their clinic on a scale from 0 to 11 (11 indicating the best practice level had been achieved) • The tool took 1–3 h to complete and aimed to include all staff working within the clinic
Action Plan setting	• Gaps highlighted within the Systems Assessment Tool and clinical data report fed into an Action Plan • Action Plan was specific to each clinic • Driven by clinical staff • Includes designated roles and responsibilities
STRIVE coordinator	• Employed through STRIVE • Five coordinators employed to work with participating sites • Worked in partnership with any existing, regionally based sexual health roles who were employed by health departments or community controlled services • Maintained regular contact with participating clinics through 3 monthly phone calls • 6 monthly face-to-face clinic visits involved delivering clinical activity reports, undertaking systems assessments and creating action plans
Health Promotion funding	• STRIVE provided clinics with a one-off $2000 payment • Payment was to be used toward an activity designed to encourage young people into the clinics for STI testing
Clinic incentive payments	• Individual clinics paid per test done and in relation to overall improvement toward meeting STI best practice targets • Money could be used as desired by each clinic

## Methods

### Setting

Communities involved in the STRIVE trial were classified by the Australian Bureau of Statistics as “very remote” [10, 11]. They were located in remote Western Australia (WA), Far North Queensland (QLD) and both the Central Australia and Top End regions of the Northern Territory (NT). Each community has a primary care centre administered either through the respective State or Territory government, or as an Aboriginal Community Controlled Health Service (ACCHS). Each ACCHS is governed by a representative board comprising local Aboriginal community members. Clinics are typically staffed by Aboriginal health workers and registered nurses with support from resident or sessional general practitioners, and visiting specialist and allied health services. Aboriginal health workers are trained clinicians, often from the community in which they work. The clinical workforce in remote communities is recognised to have a high level of staff turnover [12]. The clinics deliver health care in a complex environment, with multiple competing priorities including a high burden of chronic disease [13].

### STRIVE CQI program

The STRIVE trial examined the impact of CQI on the delivery of sexual health services within clinical settings. Clinics in STRIVE were randomly assigned to commence the CQI program in either year one, two or three of the 3 year trial. The program was based on a feedback cycle with the following components: (i) six monthly clinical data reports designed to provide clinic staff with ongoing information on their STI testing coverage; (ii) annual systems assessment to measure and describe the processes of sexual health care delivery within the clinic; (iii) action planning, to develop and guide changes to service delivery, in response to the systems assessment and clinical data reports; (iv) CQI support by a trial coordinator via six monthly face-to-face visits and regular phone calls; (v) health promotion funding consisting of a one-off $2000 payment dedicated to activities aimed at increasing clinic visits for STI testing; and (vi) incentive payments to clinics based on progress toward meeting STI best practice targets. Details of the STRIVE CQI components (summarised in Table 1) have been published previously [9]. The best practice targets included screening 80% of the resident population annually, treating 95% of symptomatic patients at the time of consultation, and retesting 80% of those treated within 3 months [9]. The trial ran from 2010 to 2014, with all but one (67/68) clinic completing the trial as detailed in the study protocol [9]. During the trial, four trial coordinators delivered 270 in-person visits to remote clinics and a further 340 phone calls to discuss components of the sexual health CQI program. Overall STRIVE results are yet to be published, however preliminary data showed an increase in STI testing rates, with testing in males increasing more than females [14].

### Data collection

Face-to-face semi-structured interviews, conducted by STRIVE coordinators (BH_PhD_ and LG_RN_), both trained in qualitative interview methods, took place within participating clinics at a time that was convenient for staff. Interviews lasted approximately one hour, were conducted in a private place within the clinic and were audio-recorded and transcribed verbatim. Interviews were conducted in two waves (2011–2012 and 2013). Separate semi-structured interview guides were developed, piloted and refined for each wave. During wave 1, interviews explored the testing and management of STIs and findings have been published [15, 16]. At clinics which had been assigned to CQI implementation for more than one year, participants were also asked about the components of the program. Interviews during wave 2 differed to wave 1 and focused on perceptions of CQI, the STRIVE trial and the impact of each component of the STRIVE CQI program (see Additional file 2).

### Study participants

Between 2011 and 2013, 41 semi-structured interviews were conducted with health staff at 22 clinics participating in STRIVE. A purposive sampling strategy – defined as selecting participants based on their level of knowledge and relevance to the topic being studied [17] – was used to select clinics and research participants within clinics to ensure a wide representation was included. Sampling was based on location of the clinic (Western Australia, Top End or Central region of the Northern Territory, or Far North Queensland), staff clinical role (Aboriginal health worker or registered nurse) and gender. Where possible, both a registered nurse and an Aboriginal health worker were interviewed to ensure equal representation. Thirteen clinics were visited in the first wave and 16 in the second (overlap of seven clinics). Interview participants consisted of registered nurses (*n* = 31) and Aboriginal Health Practitioners (*n* = 10). Sampling was impacted by multiple factors, including travel constraints, and staff availability at the time of interview. Despite this, data were collected until no new information about any interview theme was forthcoming, and ‘data saturation’ [18] was reached.

### Data analysis

Interview transcripts related to CQI from both waves were checked for accuracy against recordings and re-read several times for familiarisation prior to thematic coding [18] using QRS NVivo© (Version 10), a qualitative management and analysis program (QRS International PTY Ltd., Melbourne, Australia).

Thematic analysis consisted of two stages. First, a codebook was developed using inductive (codes from initial reads of the interview data) and deductive (codes from existing literature) approaches to data categorisation. The codebook was reviewed by BH, LM and SB and applied to all transcripts. Codes were classified into specific themes relevant to this particular study, in particular the STRIVE CQI program. Transcripts were then coded to the main themes relating to each of the six CQI components Secondly, interview transcripts were then re-read and re-coded to fit the normalisation process theory (NPT) framework illustrated in Table 2.

**Table 2:** Normalisation Process Theory components (adapted from Murray et al. 2010) [19]

Construct	Component	Interpretation
Coherence *The meaning of the intervention for interviewees*	Differentiation	How participants felt the trial procedures differed from routine practice
	Communal Specification	Did participants have a shared understanding of the trial procedures aims
	Individual Specification	Did participants have their own understanding of the trial procedures aims
	Internalisation	Did participants have an understanding of the importance and value of the trial procedures
Cognitive Participation *The level of engagement in the intervention by interviewees*	Initiation	Were participants willing to push the trial ideas forward
	Enrolment	Did participants work together to make the trial succeed
	Legitimation	Did the participants feel the trial was worthy of their time
	Activation	How likely participants were to sustain the actions within the trial procedures
Collective Action *The effort interviewees made to make the intervention work*	Interactional Workability	How trial procedures affected the work of participants together
	Relational Integration	The knowledge that builds accountability among participants
	Skillset Workability	Were the trial procedures suitable to the skillset of participants
	Contextual Integration	How compatible the trial was with existing policies, work practices or guidelines
Reflexive Monitoring *The judgement given by interviewees*	Systematisation	How effective the trial procedures were for participants
	Communal Appraisal	How groups of participants evaluated the trial procedures
	Individual Appraisal	The personal relationship participants had with the trial procedures
	Reconfiguration	Can the trial procedures be modified or adapted based on the experience of participants

### Normalisation process theory

During the second stage of data analysis, normalisation process theory (NPT) was used as an analytical framework to examine the data further. We used NPT to gain an understanding of the underlying effectiveness of the implementation and subsequent normalisation of the STRIVE CQI program [19]. NPT was developed to facilitate an understanding of complex health care interventions [19] in regard to how well they have been implemented, and normalised and integrated into clinical routine practice. NPT makes use of four analytical categories: coherence, cognitive participation, collective action and reflexive monitoring (Table 2). These categories frame the examination of factors which may have had an impact on the success of an intervention, including personal attitudes, values and the context into which the intervention is being introduced. The second phase of coding was reviewed by BH, SB and LM prior to final agreement on the framework.

### Ethical issues

Ethical approval for the STRIVE trial, including the research reported here, was obtained from UNSW Sydney, Central Australian Human Research Ethics Committee, Human Research Ethics Committee (HREC) of Northern Territory Department of Health and Families and Menzies School of Health Research, Western Australian Aboriginal Health Ethics Committee, Cairns HREC, Western Australian Country Health Service Board Research Ethics Committee. Ethical principles adhered to throughout the trial included voluntary participation, informed written consent and confidentiality and anonymity through the use of participant codes. Written consent was obtained from participants to publish quotes. Participants were not reimbursed for their participation.

## Results

Initial level thematic coding showed that the CQI components differed in perceived impact on normalisation and integration of the STRIVE CQI program. Analysis using NPT identified multiple factors perceived by health centre staff as having presented either an enabler or barrier to the integration and normalisation of the CQI program (Table 3). Participant perspectives on each component of the program are described below.

**Table 3:** Factors that enable or create a barrier to the normalisation of STRIVE sexual health CQI components

STRIVE Component	Enabler	Barrier
Clinical data report	• Data specific to each clinic • No clinic specific data pre STRIVE • Clearly highlighted gaps and improvements • Directly translatable into clinical activity • Face-to-face delivery of reports	• High staff turnover decreased relevance of the data • High staff turnover meant that some interviewees had no recall of the data reports • Data quality was questioned • Some interviewees felt the reports were difficult to interpret • Clinic data overload (too much data) • Competing clinical demands and a busy clinic
Systems Assessments Tool	• Tool aligned with existing CQI process • Tool was familiar to interviewees • Promoted reflective practice • Clearly highlighted gaps and improvements • All of staff involvement	• High staff turnover and yearly SAT meant that some interviewees had no recall of the tool • Subjective nature of scoring system created less engagement • Some clinic systems were unable to be altered by clinic based staff, therefore relevance of the tool was questioned • Tool not understood easily by all • Tool was very lengthy and felt to be repetitive
Action Plan setting	• Action plan translated the data and systems assessment into clinic specific goals • Provided a clear framework • Decreased manager workload	• High staff turnover meant some interviewees had no recall of the Action Plan • Competing clinical demands decreased the workability of the action plan • Limited engagement in the plan by some visiting sexual health support staff
STRIVE coordinator	• Clear understanding of difference in roles between STRIVE coordinator and existing regional sexual health support staff • Regular face-to-face visits • Continuity of STRIVE staff • Created accountability	• Visiting support staff ‘fatigue’
Health Promotion funding	• Encouraged staff to conduct a health promotion activity • Funding not tied to any formal reporting requirements	• Lack of clarity about the difference between STRIVE health promotion and STRIVE incentive based funding • Interviewees questioned the value of health promotion • Lack of resources (knowledge, staff, time) to utilise the funding • Difficulties in accessing the funding
Clinic incentive payments	• Funding not tied to any formal reporting requirements • Motivator for staff	• Lack of clarity about the difference between STRIVE health promotion and STRIVE incentive based funding • Difficulties in accessing the funding • Some interviewees felt ethical uncomfortable with incentivisation of their work

### Clinical data report

Participants described a range of perceived benefits that arose from receiving regular clinical data reports relevant to their own clinic. These reports enabled many participants to reflect on their clinical practice and highlighted strengths and gaps, and implement improvements in their population level management of STIs.

Prior to STRIVE, participating clinics had limited knowledge of their own STI testing and management practices at a population level. Interview narratives demonstrated that most interviewees placed a high degree of value on gaining these insights. Most participants described these data as providing insights into many aspects of guideline-driven STI care, including the age and gender of patients commonly tested, along with levels of patient follow-up (e.g. 3 monthly re-testing), thereby allowing easy translation of this data into clinical action.

*You can see the deficits and where you’ve made an improvement or what you need to target. So, for instance, we were just going in holus-bolus [all at once], testing everybody and then, upon reflection of a report … we were able to see that we were testing too many people over 35, and we really need to be focusing our attention on the proper age group, like the younger age group* (Registered Nurse, Wave 1, Top End - Northern Territory, 1-5 yrs in clinic).

Many participants reported appreciating that the data reports were presented in a visual manner. Of specific value was that the graphs could be used to demonstrate quantitative data related to each best practice clinical indicator (e.g. proportion of clinical attendees tested, proportion of individuals with a positive test result treated promptly), enabling visualisation of gaps and areas in need of improvement. Participants described understanding the information contained in the reports very easily, and did not require specific training to interpret the data presented in the reports.

Some participants explained that they were able to translate indicators presented in the reports directly into their clinical practice, and make changes accordingly.

*I was very disappointed to see that, in terms of re-testing, we got zero out of seven or something. So, while … it’s always hard to get people back in, it’s disappointing to see that none of the seven people who obviously, in that period last year, were positive, did get a three months retest. … As an individual provider, you reflect … about the number of three-monthly tests. … You think, “We did particularly badly there so maybe we should pay a bit more attention to those red recalls there* [indicating reminders within electronic medical recalls] *and attend to those”* (Registered Nurse, Wave 2, Western Australia, 1-5 yrs in clinic).

*It’s important because you need to look at data to see how effective your clinic’s been or how much you’ve screened, how much you’ve treated* (Aboriginal Health Practitioner, Wave 1, Central Australia – Northern Territory, < 1 year in clinic).

However, participants also discussed barriers to normalising the use of the clinical data reports in day-to-day practice. For example, many remote communities experience high staff turnover leading to transient staffing, which was perceived by many as reducing the positive impact of clinical data reporting as newer or temporary staff members were unable to personally relate to the data being presented (as they often were not working at the clinic during the time of the data being collected).

*Because I travelled in this last eight years, I’ve travelled so much and so frequently* [as a transient staff member], *it’s sort of been a bit hard for me at times to, to focus on having a look at those statistics* (Aboriginal Health Practitioner, Wave 2, Central Australia – Northern Territory, < 1 year in clinic).

However, this barrier diminished over time. By the second wave almost all participants were able to discuss the reports and their impact in some detail.

The perceived accuracy of the data being presented was another barrier to the integration of clinical reports into daily practice. Clinical data reports developed by STRIVE presented data that had been extracted from participating clinics’ electronic medical records, as well as analysis of laboratory data. For example, one participant questioned the accuracy of this data, in terms of population numbers, which led to diminished personal engagement in the process.

*I think they’re good but for me personally there’s been some discrepancy with the population numbers, with those reports* (Registered Nurse, Wave 1, Central Australia – Northern Territory, > 1–5 years in clinic).

Another participant felt that data inaccuracies might occur due to the manual nature of some data entry, and the many ways data can be extracted.

*I think at the beginning there was some problem with how we were recording things and how things were being looked up. ‘Cause* [electronic medical record name deleted] *is a many-headed monster and you can input things in a lot of different ways. And I think the way they were being searched for wasn’t exactly the way they were being put down. So the numbers weren’t representative of what was actually happening.* (Registered Nurse, Wave 1, Top End Northern Territory, < 1 year in clinic).

Other participants felt that the impact of the data reports was lessened due to an overload of competing data from other health areas.

*To be brutally honest, we get so much data that you’re overwhelmed with data. We get massive reporting from CQI auditing. We get reporting from KPIs* [key performance indicators]. *We get STRIVE data. We get reports from rheumatic heart data. We get so many different reports that, you know, basically you’re doing your job to the best of your ability so the data … I guess takes the back seat* (Registered Nurse, Wave 1, Central Australia, Northern Territory, 1-5 yrs in clinic).

Competing clinical demands also meant that some participants felt overwhelmed and unable to focus on the data reports.

*I think they’re* [data reports] *interesting and, in an ideal world, I would pay more heed and spend more time … but, in a clinic like* [clinic name deleted] … *there’s no time for more than the bare essentials.* (Registered Nurse, Wave 1, Western Australia, 1–5 years in clinic).

### Systems assessment tool

The STRIVE Systems Assessment Tool was based on experience from CQI programs in other areas of health [7], so was familiar to many participants. This familiarity increased the level of integration of the tool into staff work practices.

*I think that’s* [the Systems Assessment Tool] *appropriate. It’s usable and you go to any workshop and they use that as a tool, so we’re all used to filling it out … It comes fairly easily* (Registered Nurse, Wave 1, Western Australia, 1-5 yrs in clinic).

Several participants also valued the reflective practice which the tool created by highlighting strengths, gaps and areas in need of improvement. In addition, many participants felt the involvement of all clinic staff facilitated team discussions, which allowed staff to learn from each other and instilled a shared commitment to both the tool and STI control, contributing to the likelihood of it being integrated and normalised into daily practice.

*It’s* [the Systems Assessment Tool] *really helped us review ourselves … if we’re going to rate ourselves at five this time around, we take some recommendations and try to improve* via *quality improvement. And then perhaps next review we find ourselves a little bit on top of what we were previously so I mean I think it was really a good tool to motivate us to improve* (Registered Nurse, Wave 2, Far North Queensland, > 1–5 years in clinic).

*The audit [systems assessment tool] has made us all think about that* [STIs]*, especially… if you are inexperienced … there’s so much you can learn from those audits* (Registered Nurse, Wave 2, Central Australia - Northern Territory, 5-10 yrs in clinic)*.*

Participants valued the ability of the tool to quantify changes to systems over time, with some revealing that they liked to watch their scoring numbers improve, showing they were engaged in the process.

*We’re starting to really watch, to notice the numbers* (Registered Nurse, Wave 2, Far North Queensland, > 1-5 yrs in clinic).

Barriers to the normalisation of the Systems Assessment Tool were also mentioned by participants. High turnover of staff meant that some participants had no awareness of the tool, or even of similar tools. In addition, the systems assessment required that participants self-rate the clinic on a scale from 0 to 11. Some participants, both registered nurses and Aboriginal Health Practitioners, were critical of the subjective nature of this scoring, leading to disengagement in the process. However, this perception was less salient in the second waves of interviews.

*I think it’s a bit hard because clinics that have nurses flying in and out. It’s really hard because some people can make things up. Some people can say it’s an eight, when another one can say, “That’s bullshit. That’s probably a four,” you know. So if you have those sorts of issues* [disagreement in scoring] *in there. I mean you can bring them* [agreement on scoring] *out but how reliable they are, or how honest or real they are, is another question* (Aboriginal Health Practitioner, Wave 1, Central Australia – Northern Territory, < 1 yr in clinic).

*I’m not sure whether ambiguous is the right word but … It’s self-rating what you do and how you do it … if somebody else come and said, “Oh, this is how you’re doing,” you’d see, but when they ask you to self-rate how you feel that you’ve done it’s really difficult for it to be put it into perspective I s’pose* (Registered Nurse, Wave 1, Central Australia – Northern Territory, > 5–10 years in clinic).

A few participants stated that the tool was too lengthy. As a result, one participant suggested that scores may be rushed toward the end of the assessment and might not truly reflect clinical practice. Additionally, some felt frustrated by the tool as certain aspects of the clinic, such as the physical structure of the clinic building or the number of male staff, could not practically be altered or otherwise improved, or were subject to staffing constraints. Seeing little or no improvement in these aspects from year to year led to disengagement with the process. Another participants reported that following the assessment, feedback mechanisms from the clinic level to management may not have been effective, or were too slow to prompt change between assessments.

*But the trouble is we’re struggling with lots of things so we don’t ever feel like we really improve things. You know what I mean? Like you can say you’re a seven at something and really next year you’re gonna be the same. It’s frustrating because it’s all about the workload though … We have the same issues every year. They never get any better…* (Registered Nurses, Wave 2, Central Australia – Northern Territory, 5-10 yrs in clinic).

### Action plan

Most participants placed value on the action plans because the feedback mechanisms created from reviewing their clinical activity reports and the Systems Assessment Tool could be directly translated into clinic-specific goals and consequent actions. Interviewees also felt the plan provided the team with clear goals and decreased the workload of clinic managers by allowing staff to take ownership of the plan and self-direct work.

*I think that’s good …‘cause it gives us a bit of a structure, like a framework of what we’re trying to achieve. And then it’s just a reference for people to go back and think, “what is it actually we're wanting to do?” you know. And … if there’s new people coming in, then they can just have a look at that and see what we’re trying to achieve* (Registered Nurse, wave 1, Western Australia, < 1 yr in clinic).

*I reckon any action plan setting’s good ‘cause once you’ve got goals set, you’re always doing, working towards something* (Aboriginal Health Practitioner, wave 1, Top End – Northern Territory, 10+ yrs in clinic).

Once again, high turnover of staff limited the normalisation of action plans, with some participants not aware that an action plan had been created, most often because they were new to the service or not personally involved in working on a past action plan.

Competing clinical demands had an impact on the level of workability of action plans. Many participants felt that actions were often not achieved due to a busy clinical environment.

*Action plans are very effective if you have the staff. You know, it depends. We cover [community name] as well. We need dedicated staff* [to work on the action plan goals]*. You need the time. You need the effort and the resources* (Aboriginal Health Practitioner, Central Australia – Northern Territory, Wave 1, < 1 yr in clinic).

Also, while action plans encouraged accountability through designated roles and responsibilities, completion of actions depended on staff in these roles being engaged in the process. This was particularly evident with visiting and regional support staff, who were often disengaged in action plan setting but were allocated roles by participating health centre staff.

*Some of that* [goals not being actioned]*… is because some of it sits with outreach* [a regional support role] *and we never hear from her. Because it was multi-team, it wasn’t just our little, local plan. … Some of it relied on the outreach, which I think is appropriate, however none of that’s [the actions] happened* (Registered Nurse, Wave 1, Far North Queensland, 10 + yrs in clinic).

### STRIVE coordinator

The majority of participants described the important role of the STRIVE coordinators in maintaining momentum for sexual health CQI in remote clinics. In particular, interviews conducted as part of the second wave mentioned the value of the continuity provided by the STRIVE coordinator and the regularity of visits as helping to maintain rapport with clinical staff.

The regularity of contact created accountability and led to action among participants as they knew they would receive regular updates on their clinical activity. The feedback provided as part of the accountability process changed their clinical practice by increasing opportunistic sexual health screening. In addition, the regularity of face-to-face visits and consequent rapport led to interviewees feeling that they could call the STRIVE co-ordinator (who was experienced in remote sexual health) for advice when uncertain in regard to clinical matters.

*I do think face-to-face is heaps better than reading something, and then you can also put a face to these names. And if you’ve got a problem, “Oh yep, I’ll give you a ring.” Yeah. And I definitely think face-to-face is effective. Especially out here ‘cause we get so much information bombarded at us. If you can put a face to the stuff it’s much better* (Registered Nurse, Wave 2, Top End – Northern Territory, < 1 yr in clinic)*.*

*I think it’s enhanced our practice … you know you’re accountable for those things so you have to do it. So it makes you think about which way you should be doing it, or what you should be doing … and it makes you, every time somebody comes into the room, you look at ‘em and you think, “I wonder if I’ve done an STI on that person recently?” or whatever* (Registered Nurse, Wave 2, Central Australia – Northern Territory, 5-10 yrs in clinic).

Despite these benefits, some participants perceived that there were too many regionally based support programs (e.g. nutrition, health promotion, eye health) visiting the clinic. Fatigue associated with these program visits reduced the degree to which participants felt engaged with the STRIVE coordinator and CQI program.

*I think sometimes we feel a bit overwhelmed because, you know, you might get a sexual health team visit or a STRIVE team visit one week and then the next week we’ll have someone from chronic disease come out and say, “Hey, you’ve gotta get on top of your diabetics and do all this sort of stuff.” And then the next week it might be rheumatic heart disease, you know. So, “Oh do you guys realise that this is how many per cent?” you know. “Oh, you know, you’ve gotta be doing this. You’ve gotta be doing this. You’ve gotta be doing this,” and it’s sometimes hard* (Registered Nurse, Wave 2, Top End – Northern Territory, < 1 yr in clinic).

### Health promotion funding

Most participants appreciated the one-off AUD$2000 health promotion funding not being tied to any formal reporting mechanisms, in contrast to most routine funding. This flexibility increased the value of this component and therefore the level of integration. In addition, many participants felt that funding encouraged staff to conduct a health promotion activity that might otherwise not have happened.

*It has been excellent for [community name]. We’ve done two targeted screenings to the young people in that age group. We bought two iPods to start with - one for women and one for men within that age group. If they got checks, they went into the draw to win* (Registered Nurse, Wave 1, Central Australia – Northern Territory, 1-5 yrs in clinic).

However, there were very few enablers which helped normalise the health promotion aspect of the STRIVE CQI program. Instead, a lot of participants described multiple barriers to normalisation. Interviewees were confused about the difference between the two types of payments – the AUD$2000 toward a health promotion activity, and the incentive payments linked to clinical activity – offered through STRIVE. In addition, some participants were unclear about the process to access the health promotion activity funding.

*The big thing I’ve had with the money is how to access it* (Registered Nurse, Wave 2, Far North Queensland, 1-5 yrs in clinic).

Some participants also explained that they lacked the specific skills required to plan a health promotion activity, while others felt the clinic lacked the work force needed to implement an activity.

*We don’t know what good health promotion is here. We don’t want to just go and spend the money on rubbish … I think that it’s good that the money’s there but … I find it a wee bit frustrating ‘cause I’m not health promotion and I don’t think I ever will be* (Registered Nurse, Wave 1, Western Australia, 1-5 yrs in clinic).

Others did not believe in the value of health promotion activities. Some questioned the merits of staff time and clinic funding being spent on health promotion when more resources were required within the clinic.

*I think it’s a good idea short-term, but I think that money’s better spent on … creating positions in communities for sexual health workers to run sustainable programs* (Aboriginal Health Practitioner, Wave 2, Central Australia – Northern Territory, 1-5 yrs in clinic).

### Clinic incentive payments

Some services were yet to receive clinic incentive payments at the time of the interview, so their views were more hypothetical than practical. Some participants placed high value on the motivation which can be created by incentive payments and what the money could be used for. These participants also valued that these payments were not tied to any formal reporting requirements.

*I think that the incentives are good because some clinics don’t have any funding at all and the money will go towards running sexual health days or men’s health days, women’s health days … it’s beneficial for the community and the clinic* (Aboriginal Health Practitioner, T1, Central Australia – Northern Territory, < 1 yr in clinic).

As identified in relation to the health promotion funding, many participants were uncertain about the process involved and the differences between the two funding opportunities (health promotion vs. incentive payments). This lack of knowledge and uncertainty meant that this aspect of the program was not normalised for many interviewees.

Many participants also expressed concerns regarding the incentivisation of health care, creating an addition barrier to integration and normalisation.

*I don’t believe in financial incentives. I think it should be best practice. I really do. I just think we’re creating such a culture of, you know, we can only do things if there’s some sort of monetary gain and … that shouldn’t be what drives us, should it?* (Registered Nurse, Wave 1, Top End – Northern Territory, < 1 yr in clinic).

## Discussion

This study is the first investigation of primary health care providers’ perspectives on CQI strategies for sexual health service delivery in remote communities in Australia. We have identified a range of factors influencing the level of integration and degree of normalisation of the CQI program within routine clinical practice. As the program was delivered in the context of a trial, this analysis provides lessons regarding the future scalability, transferability and sustainability of the CQI program. Participants reported the greatest benefit arose from the clinical data reports. Three other components – the action planning, Systems Assessment Tool and the STRIVE coordinator role – were also perceived by staff interviewees as adding value to the program but with variable impact on practice. The two funding related components – dedicated funding for health promotion and clinic incentive payments – were described by participants as having little or no sustainable impact on clinical STI testing and management.

Use of the NPT framework highlighted a range of factors which assisted with the implementation of the STRIVE program. Participants indicated that the provision of clinic specific data reports and participation in action planning were perceived as high value activities, and encouraged and enhanced personal engagement with the study’s aims of improving STI testing and management. They perceived that these two components changed their clinical practice by increasing their likelihood of testing clinic attendees opportunistically. Regular face-to-face visits and follow-up phone calls by the STRIVE coordinator were described as fostering accountability among staff and encouraging them to work toward common goals, particularly as they knew STI testing rates would be discussed during the next visit or phone call. Aspects of the STRIVE CQI program were also easily integrated into the existing clinical processes. Interviewees explained that the Systems Assessment Tool was a tool many staff were familiar with, and that the clinical data reports provided data that was in line with clinical guidelines and relevant to day to day work. However, integration of the Systems Assessment Tool would be improved if it was shorter and the scoring system was better explained (although the reported ease of scoring did improve over time).

Many studies examining health care delivery in remote clinics acknowledge staff turnover as a consistent barrier to the delivery of best practice health care [15, 20]. We also found that high staff turnover impacted the extent to which components of the STRIVE program were normalised by staff. For example, some participants reported that staff were unaware of certain components (e.g. action plan, incentive payments); others reported feeling disengaged from CQI processes due to a lack of ownership of the care being delivered as a result of being new to the clinic or being a temporary staff member. Staff turnover is difficult to overcome and has been described as a barrier to other CQI programs in remote Aboriginal health centres [6, 21]. Barriers associated with high turnover may require systemic organisational responses. Within the context of sexual health CQI, the impact of staff turnover may be reduced by integrating CQI activities and awareness within orientation programs for new staff, gaining greater support from all levels of management and encouraging regular discussion among staff of goals highlighted within the action plan setting.

Competing clinical demands were commonly described by interviewees as a barrier to normalising CQI activities in daily practice. This is consistent with findings from other studies [8, 21, 22] and competing demands impact CQI programs as well as the delivery of guideline-driven care in many remote health centres [15]. Participants felt that competing clinical demands meant they were unable to act on many goals within the action plans, received too much data from competing programs, and experienced ‘visiting program fatigue’. Each of these factors reduced the level of normalisation of the CQI program. Health care in Aboriginal medical services has been described as being more complex, in terms of problems managed per consultation, than health care delivered within general practice in other settings [13]. Innovative methods that potentially address the competing demands in Aboriginal health settings include provision of dedicated clinical time for sexual health CQI, allocation of clinic staff to a specific focus on sexual health (e.g. responsibility for monitoring testing rates, follow-up of retesting, contact tracing and treatment), more regular reminders though face-to-face visits from regionally based sexual health support staff, phone calls or other forms of contact, and increased integration of sexual health within broader health centre CQI frameworks.

The length of time which the clinic has been participating in more general CQI programs also appears to impact the degree of normalisation and acceptance of the program. Our study found that some participants distrusted the data presented in the clinical data reports, did not engage with the rating system used during the systems assessment tool, or were simply unaware of some of the components of STRIVE. However, many of these concerns were mitigated between the first and second waves of interviews. This finding is consistent with other studies, such as CQI programs aiming to enhance adherence to the delivery of guideline driven care for type 2 diabetes in Aboriginal medical services [23, 24].

There are several limitations to this study. First, despite making every effort to conduct purposive sampling, we were bound by the practicalities and constraints of work in remote areas, which meant that interviewers could not travel to all selected health centres. Second, this is a qualitative study with a small, non-random sample size, and as such findings should not be viewed as generalizable to all remote settings. Third, our decision to use NPT as an analytical framework was made after the interviews had been conducted, potentially limiting the degree to which it could be applied. In particular, our interviews with participants were not directly guided by the theory. However, the literature suggests that this model can be used with existing data [25] and we believe this framework allowed us to effectively analyse participant perceptions regarding normalisation.

## Conclusion

Across all components of the program, the clinical data reports had the highest degree of integration and normalisation. The action plans, systems assessment tool and the STRIVE coordinator role all complemented the data reports and allowed these components to be directly translated into clinical activity. Conversely, the two types of funding – health promotion and incentive payments – had low levels of integration and normalisation. To overcome threats to the integration of sexual health CQI, programs need to align with existing clinical guidelines, be easily translated into direct clinical activity and have managerial support. To increase normalisation of the program, sexual health CQI should align with broader clinic CQI frameworks and promote staff engagement and accountability through providing clinic-specific data and regular face-to-face meetings.

## Additional files

Additional file 1: COREQ checklist. (DOCX 16 kb)

Additional file 2: STRIVE Interview Guide. (DOCX 50 kb)

## Abbreviations

ACCHS: Aboriginal Community Controlled Health Service
AUD$: Australian Dollar
CQI: Continuous Quality Improvement
HREC: Human Research Ethics Committee
KPI: Key Performance Indicator
NPT: Normalisation Process Theory
NT: Northern Territory
QLD: Queensland
STIs: Sexually Transmitted Infections
STRIVE: STIs in remote communities, improved and enhanced primary health care
WA: Western Australia
