# Supplementary material for: Perspectives of primary health care staff on the implementation of a sexual health quality improvement program: a qualitative study in remote aboriginal communities in Australia
Source: BMC Health Serv Res. 2018 Apr 2;18:230. doi: 10.1186/s12913-018-3024-y (PMC5879735; doi:10.1186/s12913-018-3024-y)
Supplement: Supplementary file 2 — STRIVE Interview Guide. (DOCX 50 kb) [file 12913_2018_3024_MOESM2_ESM.docx]

##
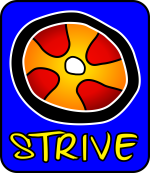
 STRIVE In Depth Interview Guide

## Year One Services

**Introductory statement**

*Acknowledgement of traditional owners and thank participants for time and input*.

My name is XX and I am here today as part of the STRIVE project. I work for XX, and part of my role is to support the delivery of sexual health services through STRIVE, and to start this process I need to understand what you think about sexual health in your clinic. I hope to hear your views on what is currently being done well within your service and what could be done better regarding the management of sexual health. It is expected that this interview will take XX mins.

The focus of STRIVE is bacterial STIs (chlamydia, gonorrhoea and trichomonas) in the 16-34 year old age group. The aim of the interview is to get your opinions about sexual health service delivery in your clinic in your own words.

To recap what we want to achieve, STRIVE aims to support services to:

- Offer STI screening to those aged 16-34 at risk of STIs
- Treat quickly – symptomatic STI infections treat immediately, asymptomatic infections treat within 7 days of the positive result being received from the laboratory
- Test for re-infection at 3 months
- Contact referral – for people with STI infection, test and treat partner/s

There is no right or wrong answers. The interview is confidential and you will not be identified.

If you agree to participate, I will be recording the interview. I am recording the interview so that I do not have to write everything down and to make sure I accurately capture your opinions and ideas.

*Signing of consent forms – pre-sign witness section (go through main points here)*

If I could start by asking you some questions – your gender, your age, your current position and role and how long you have worked in remote primary health care and how long you have worked in your current position. This information will not be recorded on the tape.

*Interviewer to ensure face sheets completed and turn on the recorder.*

**Questions:**

1. **Who currently gets tested for STIs at your clinic?**

*Probes:* When are you testing? Who are you testing? Age range? Why are you testing this group(s)?

*Prompts:* symptomatic, asymptomatic - women’s only week, well women’s week, papathon, football carnival, well men’s screening, well men’s week, men’s only time.

1. **Which groups of young people do you think are not accessing your clinic?**

*Prompts:* high risk, young men, those attending school or working away.

1. **Why? What might be some of the barriers that young people face in attending your clinic?**

*Prompts:*

**Staff/service driven:** no gender specific clinics and staff, opening hours, lack of private space for consultations.

**Patient driven:** confidentiality concerns, shame, not a priority.

1. **If a young person, aged under 25 years, came to the clinic, under what circumstances would you initiate STI testing?**

*Prompts:* symptoms, contact of a person with a known STI, adult health check, pap smears, antenatal, contraception, patient known to have travelled to nearby town.

1. **If a young person came to the clinic, what would prevent them being tested for STIs?**

*Prompts:*

**Staff/service driven:** staff time constraints/other priorities, staff not comfortable taking a sexual history, staff not aware of need to offer testing to young people, lack of private space, gender issues.

**Patient driven:** lack of knowledge about testing/treatment, shame/embarrassment, concerns about confidentiality, don’t want to know result/scared of the result.

1. **In terms of screening for STIs, what makes this different to offering screening, for example, for diabetes or hypertension?**

*Prompts:*

**Staff/service driven:** not knowing how to ask, not seen as a priority, knowing the patient too closely to ask, fear of offending the patient (seen to be placing judgement).

**Patient driven:** confidentiality concerns, shame.

1. **How do you get people back for treatment?**

*Prompts:* recall, dedicated staff, send reminder letters to patients, PCIS in-boxing, ringing other services.

1. **What makes it hard to get people treated quickly (say within one week)?**

*Prompts:* delays receiving lab result, delays actioning results, difficulty locating patients, understaffed, other clinical priorities.

1. **Can you describe the process for re-test?**

*Prompts:* 3 month test for CT/NG re-infection, set electronic recalls, opportunistic testing.

1. **What are the things that make it harder to do re-testing?**

*Prompts:* too many recalls (inappropriate recalls), hard to find people, understaffed, time constraints, other clinical priorities.

1. **Can you tell us about the process for contact tracing in the clinic?**

*Prompts:* designated staff, ask patient to inform/bring in their partners, paper-based register, set recalls for patients, phone/email/fax other services if contacts reside in other communities

1. **What makes it hard for the clinic to do contact trace partners quickly?**

*Prompts*: no dedicated person, patients not naming contacts/giving incomplete contact details, difficult to locate contacts, not maintaining appropriate records, staff comfort level in asking about contacts, over-familiarity with community, confidentiality restrictions, structural issues (lack of private space)

*Next, we are interested in finding out about your overall experience with being involved in STRIVE*

1. In terms of their contribution to improving STI testing and management, what is your opinion of each of the following:
2. Financial incentives
3. Regular reports
4. Systems assessment tool
5. Action plan setting
6. Health promotion funding
7. Electronic templates and recalls
8. Regular contact with the STRIVE coordinator

*Prompts:* What has had the greatest impact and what has had the least impact? Why?

1. **Can you describe any barriers you may have encountered in reaching some of the goals you have set through the action plan?**

*Prompts: Were there some action items you have been unable to action so far? What do you think has prevented the team from actioning these items?*

1. **What else do you think STRIVE could do to support you or the health service in achieving the best**
2. **Were there times you found it difficult to remain motivated about STRIVE?**

*Prompts: Describe the circumstances. Was this related to anything in particular? What are the key things that helped to sustain your interest and motivation and that of the other health staff?*

*Prompt: regular feedback, incentives?*

1. **If we were to run this study again, what do you think should or could be done differently?**

***A summary of the results will be available.***

***Thank you for your time. Your input is appreciated.***

##
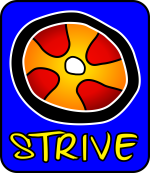
STRIVE In Depth Interview Guide - 2013

**Introductory statement**

*Acknowledgement of traditional owners and thank participants for time and input*.

My name is XX and I am here today as part of the STRIVE project. I work for XX, and part of my role is to support the delivery of sexual health services through STRIVE. To start this process I need to understand what you think about sexual health in your clinic. I hope to hear your views on what is currently being done well within your service and what could be done better regarding the management of sexual health, along with your thoughts on the STRIVE trial. It is expected that this interview will take XX minutes.

There are no right or wrong answers. The interview is confidential and you will not be identified.

If you agree to participate, I will be recording the interview. I am recording the interview so that I do not have to write everything down and to make sure I accurately capture your opinions and ideas.

***Signing of consent forms – pre-sign witness section (go through main points here)***

If I could start by asking you about your background, questions like; your gender, age and your current position. This information will not be recorded on the tape.

***Interviewer to ensure face sheets completed and turn on the recorder.***

**Questions:**

*These first set of questions are focused on what kind of things help and what kind of things hinder the testing and management of STIs within the health centre you work in.*

1. **Can you describe your understanding of best practice STI testing and management?**

*Prompts – age groups, prompt treatment, 3 month follow up, contact tracing.*

1. **Can you describe to me the barriers you and the clinic are currently facing in achieving best practice STI testing and management?**

*Prompts:*  *staffing, confidentiality and clinic layout, legalities (younger age), time constraints and other priorities.*

*Probes: (i) Can you describe how staff turnover impacts on best practice STI testing and management? (ii) Can you describe how having other clinical priorities can impact on best practice STI testing and management? (iii) Please explain to me further what you mean about reporting? (vi) Can you describe what you mean about confidentiality / lack of privacy?*

1. **Now that you’ve talked about the barriers, can you describe the kind of things that are helping you and the clinic to achieve or get close to achieving best practice STI testing and management?**

*Prompts: recall systems, AHWs, Adult health checks.*

*Probes: (i) Can you explain why having recalls helps? (ii) Why does offering a test during an Adult health check help? (iii) Can you describe why having local staff helps, are there any limitations?*

*Now I’d like to focus specifically on HIV testing.*

1. When and where would you offer HIV testing?

*Prompts: when a client has a positive PCR and being treated, antenatal screen, on history (risks)*

1. Why wouldn’t you offer HIV testing?

*Prompts: not enough time, confidentiality concerns, unsure how to ask, legalities and implications of a positive result.*

1. Can you describe the process the health centre has for managing a positive HIV result?

*Prompts: how is the health centre notified of the result? Who would tell the client? Is there anyone you could call on for support? How do you manage confidentiality?*

*Thanks, the next couple of questions are around Quality Improvement of your service and the health care your service provides more generally.*

1. **Can you describe to me your thoughts about Quality Improvement?**

*Probes: Are you aware of Quality Improvement as it relates to health care? What do you understand by Quality Improvement? How does Quality Improvement interact with your clinical work? What are the main forms of Quality Improvement you are involved in?*

1. **Can you describe any changes you have seen in the health centre that you think are related to Quality Improvement?**

*Prompts: routine auditing, routine quality reports, systems, templates,*

*Thanks, the next few questions are focused on the STRIVE trial and what has been happening within the clinic structure, management and systems since the service has been involved in the trial.*

1. **Are you aware of STRIVE, and can you describe the goals of the trial?**

*Prompts: best practice targets through quality improvement, reducing STI prevalence.*

1. **Can you describe to me what’s changed since STRIVE started, XXX health centre commenced STRIVE in xx/xx/xx?**
2. *Probes: Have there been any changes external to XX health centre? Can you describe these changes? How have they impacted on the health centre?*

*Prompts – any Medicare changes, funding, changes to the health centre’s governance structure (board, CEO, management), quality improvement.*

1. *Probes: Have there been any changes within the health centre? Can you describe these changes? How have they impacted your clinical work?*

*Prompts – staffing changes, increased awareness of data, template use, increased support from management, enhanced support from sexual health coordinators, increased awareness of treatment guidelines and importance of retesting, quality improvement.*

1. **Which of these changes do you think are related to STRIVE?** [*If answer is “none” go to question 9]*

*Prompts: data reports, templates, incentive payments, regular communication and site visits, reconnection with sexual health program, greater support from management (other things that may arise from the SAT).*

*Probes: thinking about STRIVE….*

*What do you think of [component of SHQIP]?*

*How has [component of SHQIP] made this change?*

*Ask about each component as listed below, narrative will be recorded:*

| Component | Recalls this component (yes/no) | Impact |
| --- | --- | --- |
| Systems Assessment Tool |  |  |
| Clinical activity reports |  |  |
| STRIVE co-ordinator support |  |  |
| Health promotion funding |  |  |
| Incentive payments |  |  |

1. **Can you describe how you think you or your service has benefitted from being involved in STRIVE and what has changed your clinical practice?**

*Prompts: (as above but link with clinical practice) data reports, templates, incentive payments as a motivator to test, regular communication and site visits, reconnection with sexual health program, greater support from management, other things that may arise from the SAT – link back to facilitators of STI best practice.*

1. **Do you see your service continuing to implement sexual health quality improvement procedures after the STRIVE trial and how do you see this working/not working?**

*Probe: if yes, can you describe why? Which components would be essential?*

*If not, can you describe why? What are the barriers you would see occurring?*

1. **Are you are aware that STRIVE is a research project, did that create any aspects that you liked or disliked? Can you describe these?**

*Probes: If they were not aware it was a research trial, ask what they thought it was.*

*What have you liked? What have you disliked? Can you describe this to us?*

***A summary of the results of this discussion will be available.***

***Thank you for your time. Your input is appreciated.***
